# Prior Knowledge of Epilepsy Predicts Positive Attitudes and Practices Toward Persons Living With Epilepsy

**DOI:** 10.1002/brb3.71161

**Published:** 2025-12-31

**Authors:** Nana Ama Otua Otabil, Abdala Mumuni Ussif, Francis Tanam Djankpa, Kwasi Agyen‐Mensah, Daniel Lawer Egbenya

**Affiliations:** ^1^ School of Medical Sciences, College of Health and Allied Sciences University of Cape Coast Cape Coast Ghana; ^2^ Department of Forensic Sciences, School of Biological Sciences, College of Health and Allied Sciences University of Cape Coast Cape Coast Ghana; ^3^ Department of Physiology, School of Medical Sciences, College of Health and Allied Sciences University of Cape Coast Cape Coast Ghana; ^4^ Department of Surgery, School of Medical Sciences, College of Health and Allied Sciences University of Cape Coast Cape Coast Ghana

**Keywords:** attitude, epilepsy, knowledge, misconception, practice, stigmatization

## Abstract

**Background:**

Epilepsy‐associated misconceptions may put persons living with epilepsy (PLWE) at risk of developing psychological disorders, resulting in a decrease in social contact.

**Purpose:**

Understanding knowledge, attitude, and practice (KAP) about epilepsy is essential in developing strategies to dispel these epilepsy‐associated myths and misconceptions.

**Method:**

This cross‐sectional study assessed the KAP relating to epilepsy among undergraduate university students and traders in Ghana using a structured questionnaire.

**Findings:**

Of the 532 participants surveyed, the majority were aware of epilepsy (95.3%), although 65.8% had poor/inadequate knowledge. Misconceptions were evident, as 25.9% of the participants reported that epilepsy is due to ancestral sin. Regarding attitudes toward epilepsy, 27.6% were positive, but 84.2% acknowledged societal discrimination against PLWE. A third of the participants exhibited good/appropriate practices toward PLWE. The Mann–Whitney *U* test showed a significant difference between males and females on the possession of adequate knowledge about epilepsy (male = 282.75 and female = 251.43, *p* = 0.018, *n* = 532) as well as practices toward PLWE (male = 287.34 and female = 247.17, *p* = 0.002, *n* = 532). Also, knowledge about epilepsy (*χ* ^2^ = 20.324, *p* = 0.018) and attitude toward PLWE (*χ* ^2^ = 11.880, *p* = 0.036) vary by age group, as shown by the Kruskal–Wallis test. Prior knowledge about epilepsy significantly predicted positive attitudes [AOR: 95% CI (2.794, 10.938), *p* = 0.000] and practices [AOR: 95% CI (0.675, 9.137), *p* = 0.003] toward PLWE.

**Conclusion:**

Overall, there is the need for targeted educational interventions to correct misconceptions and improve KAP about epilepsy.

## Graphical abstract

1. Possessing adequate knowledge about epilepsy could make people treat and act well towards those living with epilepsy.

2. To reduce and eliminate stigmatization against PLWE, mass media awareness campaign about epilepsy on both traditional and social media as well as community‐level information centres should be undertaken.

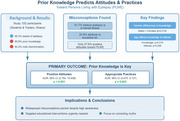

## 1 Introduction

Epilepsy is a neurological disease characterized by at least two unprovoked seizures that occur >24 h apart (Fisher et al. 2014). Epilepsy stems from functional abnormality of brain cells. It can be triggered by known factors like traumatic brain injury, brain tumors, brain infections, other space‐occupying brain pathologies, or specific congenital or genetic disorders and, in some cases, unknown causes. Despite being an age‐old condition, epilepsy is associated with a number of misconceptions, which tend to negatively affect the physical, mental, and psycho‐social well‐being of persons living with the condition.

Epilepsy, being one of the most frequently encountered brain diseases by health professionals, affects approximately 50 million people worldwide. It is one of the five commonest medical problems in Ghana (MoH 2018). It is estimated that about 270,000 people are living with epilepsy in Ghana (Mohammed Awaf 2016) with a reported prevalence of about 10.1/1000 people (Ae‐Ngibise et al. 2015). Apart from the mental and psychosocial impact and financial burden, persons living with epilepsy (PLWE) have a significantly greater percentage of physically unwell days than those without epilepsy since they suffer from frequent falls, leading to a significant impact on their general well‐being (Cardarelli and Smith 2010).

There are various beliefs about epilepsy that are not supported by substantial scientific evidence. In sub‐Saharan Africa, some people believe that epilepsy is a contagious disease that is transferred through saliva, feces, urine, and flatus during convulsion (Baskind and Birbeck 2005; Ekeh and Ekrikpo 2015).  This and other overwhelmingly negative societal beliefs about epilepsy have influenced many people to accept that epilepsy is a contagious, spiritual, and disgraceful disease (Deegbe et al. 2019). PLWE experience societal prejudice and stigmatization as a consequence of the misconceptions, negative perceptions, and defensive behavior (Elafros et al. 2015; Fiest et al. 2014; Fiest et al. 2017; Adjei et al. 2013; Krishnaiah et al. 2016). Consequently, PLWE are treated unfairly in many areas of societal institutions, including marriage, employment, and recreation (Radhakrishnan et al. 2000; Anene‐Okeke et al. 2020). The social stigma and discrimination associated with epilepsy often inflict more distress on individuals with the condition than the seizures themselves (Pandian et al. 2006). While the origins of stigma are multifaceted, ignorance about epilepsy has been identified as a key factor contributing to adverse attitudes toward PLWE (Neni et al. 2010). Victims experience social isolation or rejection that is anticipated, actualized, or perceived (Mula and Kaufman 2020). Epilepsy patients are targets for bias because they frequently experience lifelong symptoms, including seizures, loss of consciousness, and occasionally losing control of their urination or feces (Lingam 2016). Epilepsy‐associated stigma is linked to decreased marriage rates (Morrell 2002; Riasi et al. 2014), as well as decreased educational (Riasi et al. 2014; de Souza et al. 2018) and employment prospects (Chatterjee et al. 2020). The stigma associated with epilepsy exacerbates mental anguish, lowers self‐esteem and self‐efficacy, and lowers the quality of life (Newton and Garcia 2012; Herrmann et al. 2016). Also, in order to avoid being identified with the disease and stigmatized or discriminated against, people may stop looking for treatment options (Akbari et al. 2023).

Despite the ease of information accessibility these days, misconceptions about epilepsy persist even among educated persons, as they have shown poor attitudes and practices about epilepsy (Murthy et al. 2019). Gaining insight into the knowledge, attitudes, and practices (KAP) concerning epilepsy within a community is a crucial step in designing strategies to debunk myths and misunderstandings related to the condition (Deegbe et al. 2019; Braga et al. 2020). In sub‐Saharan African countries such as Ghana, university students constitute a major pool of trusted sources of information for friends, families, and their larger communities, particularly in rural and peri‐urban settings. Students possess a distinct form of influence and power (Angula NP‐I 2016). They are regarded as a knowledgeable group, which grants them significant sway over other community members. The KAP of students may, therefore, have a significant effect on shaping how their friends and families view PLWE. Consequently, we investigated the level of knowledge on epilepsy as well as attitudes and practices exhibited toward PLWE by university students and those by traders.

## 2 Methodology

### 2.1 Research Design

A cross‐sectional design with a structured questionnaire was utilized for this research. This design enabled researchers to gather information from a large number of participants at a single instance on KAP toward PLWE and also ensured the comparison of differences between and among groups.

### 2.2 Profile of Study Area

The study was carried out at the University of Cape Coast (UCC) and Abura Market, both of which are situated in the historic town of Cape Coast in Ghana. The university is located on a hill overlooking the Atlantic Ocean. Established in 1962 by the Ghanaian government, the university was initially aimed at fulfilling the demand for highly skilled individuals in the education sector, specifically for the training of teachers for science education. However, as time passed, it broadened its academic offerings to include diverse fields such as medical sciences, business administration, agricultural and consumer sciences, and natural sciences. The Abura Market is one of the main market centers in the Cape Coast metropolis where various products and services are rendered. Many hundreds of people patronize the market on a daily basis.

### 2.3 Study Population, Sample Size and Sampling Technique

The target population was UCC undergraduate students and Abura market traders (non‐students). Overall, a sample size of 532 was used in the present study. This computation was based on the Yamane formula (Israel 1992).

The convenience sampling method was used to sample respondents who were readily available. UCC undergraduate students and Abura Market traders who consented to the study were included. Prospective participants who may have suffered a seizure or anyone who has any form of mental disorder (based on verbal report) were excluded from the study.

### 2.4 Data Collection Tool and Data Collection

Data collection tools included the administration of a structured questionnaire to study participants. The validated instrument used was modified based on previous studies (Krishnaiah et al. 2016; Perucca 2014; Sinha et al. 2023). The questionnaire was written and administered in English. The test instrument was pretested to assess its reliability. For the pre‐test study, 53 participants took part. The Cronbach's alpha reliability coefficient of the test instrument (questionnaire) was found to be 0.89.

The items in the questionnaire were structured with both open‐ and closed‐ended questions. There were four sections on the questionnaires: A, B, C, and D. The first section (A) captured the socio‐demographic factors, which included age, sex, student's residential status, department, ethnicity, religious affiliation, etc. The second part (B) tested students' epilepsy knowledge, including their knowledge of the condition's symptoms and myths, whereas the third section (C) assessed their attitudes about epilepsy and PLWE. The practices that students may engage in toward PLWE were assessed in the fourth section (D). Each item had three options (Yes/No/I do not know). Some items in each domain had negative statements. For instance, items in the questionnaire include “Is epilepsy a contagious disease?” (Knowledge section), “Will you keep someone with epilepsy as a friend?” (Attitude section) and “Put a spoon in the mouth of someone with an epileptic attack” (Practice section).

During the questionnaire administration (self‐administered), any respondent who needed clarification about any part of the questionnaire was assisted by the questionnaire administrators. Data was collected from participants from March 12 to May 3, 2024.

### 2.5 Ethical Considerations

Ethical clearance for the conduct of the present study was obtained from the University of Cape Coast Institutional Review Board (UCCIRB/CHAS/2023/03). The researcher provided full information about the purpose of the research to the participants. Participants were allowed to ask questions pertaining to the study, and answers were provided. Additionally, prospective participants were informed that participating in the study was voluntary and that they could withdraw at any time in the course of the study if they so wished. Thereafter, a prospective participant's written consent was obtained. In ensuring confidentiality of respondents’ information, each questionnaire submitted had a unique code instead of participants’ full names, and the data was stored in an electronic database secured with a password.

### 2.6 Data Analysis

Statistical analysis was performed using Statistical Product and Service Solutions (SPSS) version 22 (IBM, US). Socio‐demographic data as well as participants' knowledge, attitudes, and practices were analyzed using descriptive statistics like frequencies. In scoring the sections on knowledge, attitudes, and practices, a score of 1 was given as the correct answer and a score of 0 for wrong answers, including “I don't know” responses. Categorization of levels of KAP was computed using Bloom's cut‐off categorization: < 60% classified as poor/inadequate knowledge, attitude and practice; 60–79% categorized as moderate knowledge, attitude, and practice; while 80–100% was considered adequate/good knowledge, attitude, and practice relating to epilepsy (Feleke et al. 2021). Using binary logistic regression, the factors influencing knowledge, attitudes, and practices were also identified. Crude and adjusted odds ratios as well as Mann–Whitney *U* and Kruskal–Wallis tests at a 95% confidence interval were used to test the significance of the participants’ responses in this exploratory research. A statistically significant result was considered when the *p*‐value was < 0.05.

## 3 Results

### 3.1 Demographic Characteristics of Respondents

Table 1 displays the demographic characteristics of the respondents. About 72% of the participants were 18–27 years old (reflecting the high number of tertiary‐level students in the study), with about 4% of the respondents being 58 years or older. Slightly more than half (51.9%) of the participants were females. The participants included a large proportion of students (64.5%), with about a third being traders (35.5%). The students span across various colleges within the university and across all undergraduate levels of study, with a little over 54% of the participants belonging in the College of Health and Allied Sciences (COHAS). Only 0.2% of the participants were Distant Programme students, that is, College of Distance Education (CODE). Most respondents identified as Christians (89.5%), and Akan was the dominant ethnic group examined (73.7%).

**TABLE 1:** Socio‐demographic data of respondents.

Demographic	Response	Frequency (n)	Percentage (%)
**Age (years)**	Below 18 18–27 28–37 38–47 48–57 58 and above	21 382 64 30 15 20	3.9 71.8 12.0 5.6 2.8 3.8
**Sex**	Male Female	256 276	48.1 51.9
**Occupation**	Student Non‐student (Trader)	343 189	64.5 35.5
**Students’ college**	COHAS CANS CODE CES CHLS	185 41 2 17 97	54.1 12.0 0.6 5.0 28.3
**Students’ level (study year)**	100 200 300 400	60 120 133 31	17.4 34.9 38.7 9.0
**Religion**	Christian Muslim Traditional Other	476 47 2 7	89.5 8.8 0.4 1.3
**Ethnicity**	Akan Ewe Ga‐Dangbe Mole‐Dagbani Other	392 59 18 12 51	73.7 11.1 3.4 2.3 9.6

**Abbreviations**: CANS, College of Agriculture and Natural Sciences; CES, College of Education Studies; CHLS, College of Humanities and Legal Studies; CODE, College of Distance Education; COHAS, College of Health and Allied Sciences.

### 3.2 Sources of Information on Epilepsy

Sources of information on epilepsy are included in Table 2. The majority of the participants (95.3%) had previously heard of epilepsy. The “friends and family” category was reported as the highest primary source of information about epilepsy (18.6%), followed by “radio/TV and newspaper” (15.5%), while “school” placed third with 12.8%. “Health facilities,” such as hospitals, came forth as the predominant source of information about epilepsy, while more than a quarter of the respondents (29.3%) indicated that they had heard about epilepsy from more than one of the named sources.

**TABLE 2:** Sources of information on epilepsy.

Variable	Response	Frequency (n)	Percentage (%)
**Heard of epilepsy?**	Yes No	507 25	95.3 4.7
**Sources of knowledge**	Radio/TV/Newspaper Internet Health facility Lectures/school Friends and family Social media Other Multiple	80 29 46 66 96 22 26 151	15.5 5.6 8.9 12.8 18.6 4.3 5.0 29.3

*Some respondents gave more than one response on their sources of information on epilepsy.

### 3.3 Knowledge of Epilepsy Among Participants

About two‐thirds of the participants (63.2%) had ever seen a person having an epileptic attack in the past (Table 3). While 61.7% of the participants were of the view that epilepsy is a mental condition, about 74% of them thought epilepsy is, generally, an inheritable disorder. More than two‐thirds of the participants (67.9%) thought epilepsy is not an infectious disease. The majority of participants (74.1%) did not believe that ancestors' sins contributed to epilepsy. Also, the majority of participants (88.7%) believed that epilepsy reduces the quality of one's life, hence an unhappy life. Nearly half of the participants, 51.1%, felt that persons with epilepsy cannot have a normal sexual life. The majority of participants (84.8%) thought that epilepsy could affect the education of a person, with almost half of them saying that PLWE can work properly at work. Additionally, about 40% of the participants thought that epilepsy was untreatable.

**TABLE 3:** Knowledge about epilepsy.

Knowledge items	Frequency	Percent (%)
**Have you ever observed someone having a seizure?**		
Yes No	336 196	63.2 36.8
**Is epilepsy a form of mental illness?**		
Correct (No) Wrong (Yes)	204 328	38.2 61.7
**Is epilepsy, in general, an inheritable condition?**		
No (Correct) Yes (Wrong)	139 393	26.1 73.9
**Is epilepsy a contagious disease?**		
Correct (No) Wrong (Yes)	361 171	67.9 32.1
**Do you believe that an ancestor's sin brought about epilepsy?**		
Correct (No) Wrong (Yes)	394 138	74.1 25.9
**Do you believe that having epilepsy makes one less happy in life?**		
Correct (No) Wrong (Yes)	60 472	11.3 88.7
**Can persons who suffer from epilepsy have normal sexual lives?**		
Correct (Yes) Wrong (No)	260 272	48.9 51.1
**Do you believe that epilepsy might affect an individual's capacity to learn at school?**		
Correct (No) Wrong (Yes)	81 451	15.2 84.8
**Do you believe that epilepsy is treatable?**		
Correct (Yes) Wrong (No)	321 211	60.3 39.7
**Do you believe that people with epilepsy can function properly at work?**		
Correct (Yes) Wrong (No)	270 262	50.8 49.2

**FIGURE 1:**
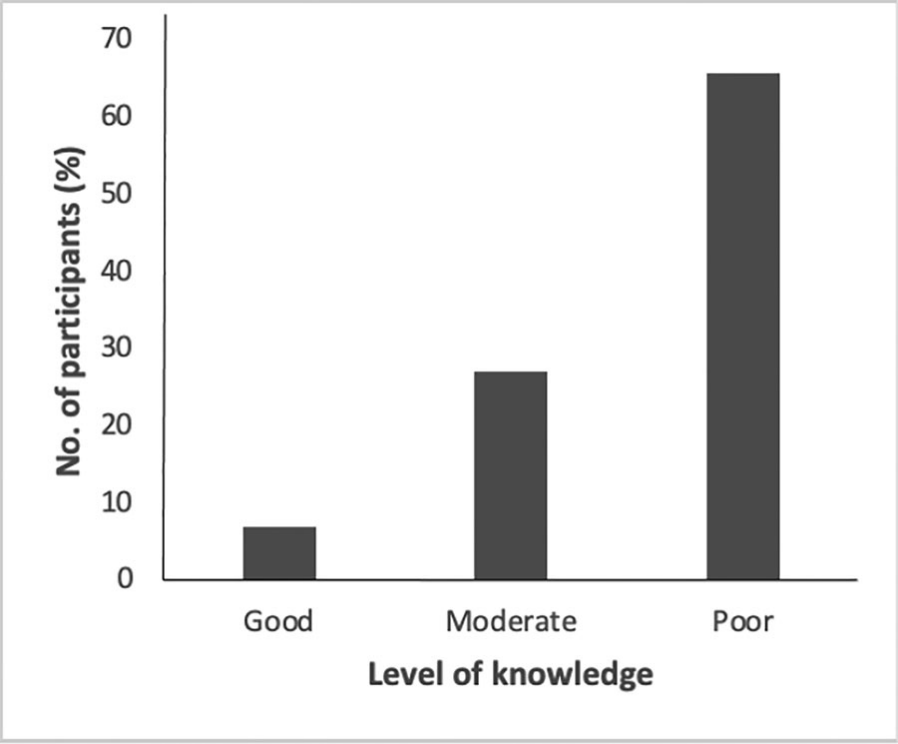
Level of knowledge of epilepsy among participants. About two‐thirds of the respondents showed inadequate knowledge (65.8%), 27.1% had moderate knowledge, and only 7.1% demonstrated a good/adequate level of knowledge about epilepsy.

Figure 1 illustrates the level of knowledge about epilepsy among the study participants. Based on Bloom's cut‐off categorization, 350 (65.8%) of the participants had poor knowledge of epilepsy while a little over a quarter of the respondents possessed moderate knowledge, that is, 144 (27.1%). Additionally, only 38 (7.1%) of the respondents demonstrated good/adequate knowledge about epilepsy.

### 3.4 Attitudes Toward Epilepsy Among Participants

The majority of participants (84.2%) indicated that people with epilepsy are faced with societal discrimination (Table 4). Similarly, 77.3% were willing to maintain a PLWE as a friend. Interestingly, however, only 14.3% of the participants said they would be interested in marrying someone with epilepsy. A little over two‐thirds of the participants (67.1%) said they would permit their kids to play with a young person with epilepsy. More than half of the participants (56.8%) were of the view that students with epilepsy should attend alternative or compensatory schools, but 85.5% of the study participants are willing to share their stationeries with PLWE. About a third of the participants (31.2%) disagreed that people with epilepsy can have successful lives. As high as 91.2% of the participants expressed an interest to learn more about epilepsy. Again, using Bloom's cut‐off point as a reference, 151 (28.4%) demonstrated negative attitudes toward PLWE, while 234 (44.0%) expressed moderate attitudes. Equal numbers of respondents demonstrated negative and positive attitudes toward PLWE (147 people, i.e., 27.6%).

**TABLE 4:** Participants’ attitudes toward PLWE.

Participants’ attitudes toward epilepsy	Frequency	Percent (%)
**Do you agree that people with epilepsy face discrimination from society?**		
Correct Wrong	448 84	84.2 15.8
**Will you keep someone with epilepsy as a friend?**		
Yes No	411 121	77.3 22.7
**Will you get married to someone with epilepsy?**		
Yes No	76 456	14.3 85.7
**Would you let your child to play with another child who has epilepsy?**		
Yes No	357 175	67.1 32.9
**Epilepsy patients need to be enrolled in alternative or compensatory schools**.		
Correct Wrong	230 302	43.2 56.8
**Would you share your stationaries like pens, pencils and books with someone with epilepsy?**		
Yes No	455 77	85.5 14.5
**People with epilepsy may live successful lives**		
Correct Wrong	366 166	68.8 31.2
**Do you want to know more about epilepsy?**		
Yes No	485 47	91.2 8.8

### 3.5 Practices of Participants toward Persons With Epilepsy

As a practice measure, we examined students’ knowledge on first aid in the management of seizures (Table 5). Participants were asked what they would do if they saw someone experiencing an epileptic attack. About two‐thirds (66.5%) of the participants would either put a spoon in the mouth of the patient or, not knowing what to do, call the doctor/nurse (84.8%), remove any object lying around immediately (91.7%), and position the patient after an attack (90.0%), and almost half of the participants will start praying (51.3%).

**TABLE 5:** Participants’ practice‐related responses to an epileptic attack.

Practices toward an epileptic attack	Frequency	Percent (%)
**Put spoon in mouth**		
Correct Wrong	178 354	33.5 66.5
**Call a doctor or nurse**		
Correct Wrong	451 81	84.8 15.2
**An Object lying around should be immediately removed**		
Correct Wrong	488 44	91.7 8.3
**Position the patient safely after an attack**		
Correct Wrong	479 53	90.0 10.0
**Start praying**		
Correct Wrong	259 273	48.7 51.3
**Apply anointing oil on the person**		
Correct Wrong	451 81	84.8 15.2
**Take the person to the church/mosque/shrine**		
Correct Wrong	429 103	80.6 19.4
**Run away**		
Correct Wrong	485 47	91.2 8.8

The nature of practices toward a person with an epileptic attack among the participants is presented in Figure 3. Close to one‐third of the respondents, that is, 170 (32%), showed positive practice on first aid management for epilepsy. Meanwhile, 94 (17.7%) had negative practices/dispositions toward someone experiencing an epileptic fit, and moderate/neutral practices were shown by half of the participants, that is, 268 (50.4%).

### 3.6 Influence of Gender and Occupation on KAP About Epilepsy

We also investigated whether or not gender and occupation of participants affected their KAP toward PLWE. Males had higher/better knowledge about epilepsy and demonstrated more positive attitudes toward PLWE than their female counterparts. It was found that significant differences existed between males and females on knowledge about epilepsy (male = 282.75 and female = 251.43, *p* = 0.018, *n* = 532 for both groups) as well as practices toward PLWE (male = 287.34 and female = 247.17, p = 0.002, n = 532 for both groups) (Table 6). Additionally, we report that significant differences exist between students and non‐students (traders) on attitude (students = 280.10 and non‐students = 241.82, *p* = 0.005, *n* = 532 for both groups) and practices (students = 291.11 and non‐students = 221.84, *p* = 0.000, *n* = 532 for both groups) toward PLWE. Similarly, students showed more positive attitudes and practices toward PLWE than traders (non‐students).

**TABLE 6:** Influence of gender and occupation on KAP about epilepsy using the Mann–Whitney *U* test.

KAP	Factor	N	Mean rank	SD	p value
	**Gender**				
**Knowledge**	Male	256	282.75	2.049	0.018*
	Female	276	251.43		
**Attitude**	Male	256	274.88	1.671	0.217
	Female	276	258.73		
**Practice**	Male	256	287.34	1.391	0.002*
	Female	276	247.17		
	**Occupation**				
**Knowledge**	Student	343	274.82	2.049	0.089
	Trader	189	251.41		
**Attitude**	Student	343	280.10	1.671	0.005*
	Trader	189	241.82		
**Practice**	Student	343	291.11	1.391	0.000*
	Trader	189	221.84		

### 3.7 Influence of Participant's Age and Student's College on KAP Toward Epilepsy

Participants aged 28–37 years old had the highest level of knowledge about epilepsy and also demonstrated the most appropriate attitudes toward PLWE than all other age groups investigated. Regarding knowledge about epilepsy and attitude toward PLWE, significant differences exist among the various age groups investigated since *χ* ^2^ = 20.324, *p* = 0.018 and *χ* ^2^ = 11.880, *p* = 0.036, respectively (Table 7). For college affiliation of students, those from the College of Health and Allied Sciences possessed the highest level of knowledge on epilepsy and also demonstrated the most appropriate attitudes and practices toward PLWE. There were significant differences among participants on all three key areas investigated: knowledge (*χ* ^2^ = 72.745, *p* = 0.000), attitude (*χ* ^2^ = 55.321, *p* = 0.000), and practice (χ^2^ = 58.905, *p* = 0.000) when considering students’ college affiliation.

**TABLE 7:** Assessing the effect of age and students’ college on KAP about epilepsy using Kruskal Wallis test.

KAP	Factor	N	Mean rank	SD	χ^2^	*p* value
	**Age**					
	> 18	21	258.88	2.049	20.324	0.018*
	18 – 27	382	252.21			
Knowledge	28 – 37	64	341.96			
	38 – 47	30	276.27			
	48 – 57	15	306.10			
	≤ 58	20	261.68			
	> 18	21	227.50	1.671	11.880	0.036*
	18 – 27	382	258.58			
Attitude	28 – 37	64	322.13			
	38 – 47	30	277.58			
	48 – 57	15	286.37			
	≤ 58	20	249.15			
	> 18	21		1.391	3.279	0.657
	18 – 27	382	257.64			
Practice	28 – 37	64	266.47			
	38 – 47	30	289.06			
	48 – 57	15	261.13			
	≤ 58	20	262.20			
	**College**					
Knowledge	COHAS	185	210.64	2.049	72.745	0.000*
	CANS	41	161.38			
	CODE	2	116.25			
	CES	17.	134.53			
	CHLS	97	108.75			
Attitude	COHAS	185	206.15	1.671	55.321	0.000*
	CANS	41	143.60			
	CODE	2	25.25			
	CES	17	135.91			
	CHLS	97	126.46			
Practice	COHAS	185	204.63	1.391	58.905	0.000*
	CANS	41	165.27			
	CODE	2	97.00			
	CES	17	169.09			
	CHLS	97	112.91			

For practice toward PLWE, there were significant differences among the various ethnic groups examined (*χ* ^2^ = 10.627, *p* = 0.031) but not on knowledge and attitude toward PLWE. Additionally, in terms of level of studies, significant differences exist on attitude toward PLWE (*χ* ^2^ = 4.777, *p* = 0.010) but not knowledge and practice. Kruskal–Wallis test results showed no significant differences among participants’ religions on KAP about epilepsy (results not shown).

### 3.8 Determinants of Adequate/Good Knowledge About Epilepsy

In Table 8, we present the factors predicting or influencing good/adequate knowledge of epilepsy among the respondents. The logistic regression analysis showed that, if unadjusted, gender, age, ethnicity, occupation, student level, college, prior knowledge of epilepsy, and positive/good attitudes and practices toward PLWE predicted a good or adequate level of knowledge about epilepsy. However, when adjusted, only age, college, having ever seen an epileptic fit, and practice predicted adequate knowledge of epilepsy.

**TABLE 8:** Predictors of good or adequate knowledge of epilepsy.

Variable/category	COR	95% CI for COR	*p* value	AOR	95% CI for AOR	*p* value		
		Lower	Upper		Lower	Upper		
**Gender**								
Female	0.607	0.422	0.873	0.007*	1.318	0.717	2.423	*0.374*
Male	1							
**Age (years)**				0.003*				0.013*
Below 18	0.806	0.310	2.100	*0.659*	2.519	0.323	19.626	*0.378*
18 – 27	2.440	0.846	7.031	*0.099*	13.915	1.303	148.570	0.029*
28 – 37	0.623	0.181	2.142	*0.453*				
38 – 47	1.143	0.284	4.595	*0.851*				
48 – 57	0.571	0.144	2.262	*0.425*				
58 and above	1							
**Religion**				*0.132*				*0.962*
Christianity	0.583	0.288	1.180	*0.134*	1.495	0.346	6.464	*0.590*
Islam	0.000	0.000		*0.999*	0.000	0.000		*0.999*
Traditional	4.503	0.864	23.465	*0.074*	7.6x10^8^	0.000		*1.000*
Other	1							
**Ethnicity**				*0.089*				*0.535*
Akan	2.097	1.201	3.661	0.009*	1.585	0.689	3.647	*0.278*
Ewe	1.677	0.646	4.355	*0.288*	1.957	0.533	7.180	*0.312*
Ga/Dangbe	1.747	0.523	5.836	*0.364*	1.192	0.236	6.005	*0.832*
Mole/Dagbani	1.048	0.554	1.982	*0.884*	2.110	0.667	6.680	*0.204*
Other	1							
**Occupation**	0.574	0.388	0.849	0.005*	0.000	0.000		*1.000*
**Student level**				*0.099*				*0.487*
Level 100	1.998	1.011	3.949	0.046*	0.684	0.236	1.984	*0.485*
Level 200	2.014	1.027	3.947	0.041*	0.715	0.243	2.105	*0.543*
Level 300	1.074	0.409	2.821	*0.885*	0.383	0.113	1.298	*0.123*
Level 400	1							
**College**				0.000*				0.006*
COHAS	0.365	0.178	0.750	0.006*	0.612	0.198	1.887	*0.393*
CANS	0.000	0.000		*0.999*	0.000	0.000		*0.999*
CODE	0.105	0.023	0.472	0.003*	0.056	0.007	0.422	0.005*
CES	0.145	0.076	0.275	0.000*	0.306	0.138	0.679	0.004*
CHLS	1							
**Ever heard**	0.281	0.082	0.961	0.043*	0.347	0.033	3.695	*0.380*
**about epilepsy**								
**Seen epilepsy**	0.170	0.105	0.275	0.000*	5.481	2.764	10.867	0.000*
**Attack**								
**Attitude**	6.760	4.357	10.486	0.000*	5.788	2.886	11.607	*0.171*
**Practice**	3.376	1.847	6.172	0.000*	2.484	0.675	9.137	0.003*

### 3.9 Determinants of Good or Positive Attitude Toward Persons With Epilepsy

In this study, we also examined the predictors of positive attitudes toward PLWE. We found out that, if unadjusted, occupation, level of students, college, ever seen and heard about epilepsy, adequate knowledge about epilepsy, and positive practices toward PLWE significantly predicted positive attitudes toward PLWE (Table 9). Upon adjusting the odds ratio, only three of the factors investigated significantly predicted positive or good attitudes toward PLWE, that is, ever hearing and seeing someone with an epileptic fit as well as possession of adequate knowledge of epilepsy.

**TABLE 9:** Predictors of good or positive attitudes toward epilepsy

Variable/category COR	95% CI for COR	*p* value	AOR	95% CI for AOR	*p* value			
	Lower	Upper		Lower	Upper			
**Gender**								
Female	0.841	0.598	1.306	*0.323*	1.235	0.714	2.137	*0.451*
Male	1							
**Age (years)**				*0.065*				*0.757*
Below 18	0.963	0.400	2.321	*0.933*	0.879	0.143	5.417	*0.890*
18 – 27	2.513	0.906	6.972	*0.077*	1.396	0.158	12.322	*0.764*
28 – 37	1.189	0.388	3.644	*0.762*				
38 – 47	1.039	0.275	3.919	*0.955*				
48 – 57	1.100	0.267	3.096	*0.879*				
58 and above								
**Religion**				*0.787*				*0.971*
Christianity	0.779	0.428	1.419	*0.415*	1.396	0.365	5.347	*0.626*
Islamic	0.813	0.051	13.074	*0.884*	1.046	0.060	18.347	*0.976*
Traditional	0.610	0.135	2.754	*0.520*	1.7x10^8^	0.000		*1.000*
Other								
**Ethnicity**				*0.180*				*0.426*
Akan	1.659	0.939	2.929	*0.081*	1.102	0.468	2.599	*0.824*
Ewe	2.383	0.834	6.813	*0.105*	3.079	0.799	11.868	*0.102*
Ga/Dangbe	1.833	0.543	6.188	*0.329*	1.780	0.363	8.733	*0.478*
Mole/Dagbani	0.953	0.532	1.709	*0.873*	0.671	0.227	1.984	*0.471*
Other	1							
**Occupation**	0.622	0.435	0.889	0.009*	0.000	0.000		*1.000*
**Student level**				*0.139*				*0.988*
Level 100	2.037	1.086	3.820	0.027*	0.914	0.361	2.313	*0.850*
Level 200	1.904	1.028	3.526	0.041*	0.904	0.364	2.246	*0.827*
Level 300	1.692	0.705	4.064	*0.239*	0.825	0.286	2.379	*0.722*
Level 400	1							
**College**				0.000*				*0.146*
COHAS	0.368	0.184	0.737	0.005*	0.501	0.182	1.374	*0.179*
CANS	0.000	0.000		*0.999*	0.000	0.000		*0.999*
CODE	0.311	0.114	0.853	0.023*	0.394	0.114	1.361	*0.141*
CES	2.854	0.117	0.336	0.000*	0.442	0.230	0.850	*0.014*
CHLS	1							
**Ever heard**	0.148	0.050	0.436	0.001*	0.178	0.032	0.996	0.049*
**about epilepsy**								
**Seen epilepsy**	2.620	1.823	3.765	0.000*	2.114	1.164	3.842	0.014*
**Attack**								
**Knowledge**	6.760	4.357	10.486	0.000*	5.528	2.794	10.938	0.000*
**Practice**	2.288	1.443	3.625	0.000*	1.621	0.692	3.796	*0.266*

### 3.10 Determinants of Positive/Good Practices Toward PLWE

With unadjusted odds ratios, occupation, level of students, college, ever heard and seen someone with an epileptic fit, adequate knowledge about epilepsy, and positive attitude toward PLWE significantly predicted positive practices toward PLWE (Table 10). However, upon adjusting the odds ratio, it was only having ever seen a person with an epileptic fit, a form of prior knowledge, that significantly predicted positive or good practices toward PLWE [AOR (95% Confidence Interval): 3.716 (1.505, 9.175), *p* = 0.004].

**TABLE 10:** Predictors of positive/good practices towards PLWE.

Variable/ category COR	95% CI for COR	*p* value	AOR	95% CI for AOR	*p* value			
	Lower	Upper		Lower	Upper			
**Gender**								
Female	0.685	0.436	1.077	*0.101*	1.188	0.560	2.520	*0.654*
Male	1							
**Age (years)**				*0.948*				*0.993*
Below 18	1.169	0.381	3.590	*0.785*	0.000	0.000		*0.999*
18 – 27	0.840	0.243	2.904	*0.783*	0.000	0.000		*0.999*
28 –	1.176	0.275	5.025	*0.826*				
38 – 47	0.941	0.177	4.997	*0.943*				
48 – 57	0.941	0.201	4.412	*0.939*				
58 and above	1							
**Religion**				*0.997*				*0.975*
Christianity	0.918	0.428	1.970	*0.918*	0.688	0.141	3.347	*0.643*
Islamic	3.5x10^8^	0.000	0.999	*1.2x10^8^*	0.000		0.999	
Traditional	3.5x10^8^	0.000	0.999	*2.0x10^7^*	0.000			*1.000*
Other	1							
**Ethnicity**				*0.492*				*0.410*
Akan	1.145	0.537	2.441	*0.725*	1.158	0.277	4.838	*0.841*
Islamic	0.536	0.185	1.554	*0.251*	0.291	0.079	1.079	*0.065*
Traditional	2.268	0.288	17.862	*0.437*	1.397	0.132	14.784	*0.781*
Other	0.670	0.333	1.347	*0.261*	0.675	0.155	2.949	*0.601*
**Occupation**	0.431	0.275	0.678	0.000*	7.0x10^7^	0.000		*1.000*
**Student level**				0.049*				*0.602*
Level 100	3.348	1.387	8.083	0.007*	1.850	0.556	6.148	*0.316*
Level 200	2.226	1.006	4.925	0.048*	1.381	0.465	4.101	*0.561*
Level 300	1.583	0.512	4.893	*0.425*	0.744	0.193	2.869	*0.667*
Level 400	1							
**College**				0.001*				*0.387*
COHAS	0.369	0.128	1.063	*0.065*	0.851	0.215	3.365	*0.818*
CANS	0.063	0.004	1.080	*0.057*	0.155	0.007	3.296	*0.232*
CODE	0.205	0.057	0.736	0.015*	0.284	0.058	1.389	*0.120*
CES	0.216	0.100	0.467	0.000*	0.571	0.222	1.469	*0.245*
CHLS	1							
**Ever heard**	0.533	0.216	1.314	*0.172*	0.422	0.110	1.616	*0.208*
**about epilepsy**								
**Seen epilepsy**	2.814	1.785	4.436	0.000*	3.716	1.505	9.175	0.004*
**Attack**								
**Knowledge**	3.376	1.847	6.172	0.000*	2.424	0.708	8.291	*0.158*
**Attitude**	2.288	1.443	3.625	0.000*	1.664	0.717	3.866	*0.236*

## 4 Discussion

In the present study, we investigated the knowledge level, kind of attitudes, and nature of practices that university students and traders in the Cape Coast metropolis exhibit towards PLWE. In doing so, 532 participants were administered a structured questionnaire, and their responses were analyzed.

### 4.1 Sources of Knowledge of Epilepsy

The findings in Table 2 showed that the majority of the participants (95.3%) are aware of epilepsy. This high degree of awareness can be attributed to the participants' exposure to a number of informational sources, especially the media and educational institutions, as well as interactions with family and friends. This suggests that there is a relatively high level of general awareness about epilepsy in the population, which is important for fostering understanding and reducing stigma. The findings are consistent with previous studies that demonstrated a growing level of awareness of epilepsy in various populations (Ngugi et al. 2010).

The most prominent source of information (knowledge) on epilepsy among the participants was through interactions with “friends and family,” with 18.6% of the respondents citing it as their main source, implying an active level of interpersonal relationships/familial interactions in the population. About 13% cited school as their source of information on epilepsy, underscoring the importance of educational institutions in providing accurate and comprehensive information about epilepsy (Brabcová et al. 2017). Cumulatively, mass media (radio/TV/newspaper, internet, and social media) was the predominant means of accessing information related to epilepsy by the respondents, that is, 25.4%. The growing influence of the internet on health information‐seeking behavior is well‐documented (Boulos et al. 2011), and its role in epilepsy awareness is no exception. This result is in line with previous reports, which identified mass media as an effective channel for influencing how the general population perceives health‐related issues (Ngugi et al. 2010). Many respondents also reported using multiple sources for their epilepsy‐related information. The findings highlight the significant role that different channels of communication play in disseminating information and raising awareness about epilepsy; hence, leveraging these channels during public awareness creation efforts on the condition will be vital.

The number of participants who acquired information from health facilities emphasizes the important role that healthcare providers play in educating patients and their families (Millogo and Siranyan 2004). However, a lower percentage of participants cited health facilities (8.9%) as their source of knowledge on epilepsy compared to other sources. One may be tempted to attribute this result to the likely healthy state of many of the students, who constituted the bulk of the present sample and hence are less unlikely to visit health facilities frequently for epilepsy‐related information. However, among traders alone, “friends and family” remains the predominant source of epilepsy‐related information (32.4%), while health facilities placed sixth with 6.0% (not shown). The present finding, therefore, suggests a need for increased focus on epilepsy education within healthcare settings and health facility‐sponsored mass education programs, as well as improved collaboration between healthcare providers and other sources of information. Health facilities play a crucial role in providing accurate and up‐to‐date information on epilepsy, which is essential for appropriate diagnosis, treatment, and management of the condition (Ngugi et al. 2010). Overall, the results on sources of information about epilepsy showed that various sources of information contribute to the overall awareness of epilepsy among the studied population. Future research and public health initiatives should continue to leverage these channels to further enhance awareness of epilepsy and likely contribute to reducing stigma associated with the condition.

### 4.2 Knowledge of Epilepsy Among the Participants

The survey results provide valuable insights into the perceptions and understanding of epilepsy among the participants. The results demonstrate that while a majority of participants were aware of epilepsy, there were still misconceptions and stigma associated with the condition (Table 3). The majority of the participants (95.3%) had heard of epilepsy, indicating that awareness of the disorder is relatively high, which is reassuring to know. Despite this high level of awareness, as much as a third of the participants had never seen an epileptic seizure occur, which may contribute to misconceptions or lack of comprehensive understanding of the condition (Robert et al. 2005). The perception of epilepsy as a mental illness by 61.7% of the participants is indicative of prevailing misconceptions. This is consistent with earlier research, which revealed similar beliefs (Birbeck 2010). Epilepsy, which can co‐exist (as a comorbidity) with a mental health condition, is primarily a neurological disorder characterized by recurrent seizures. Additionally, many participants were unsure about the etiology or pathophysiological basis of the condition, indicating the need for further education and awareness campaigns.

The belief held by 73.9% of the participants that epilepsy is a hereditary disease is partially accurate. While some forms of epilepsy do have a genetic component, not all types are hereditary (Ottman et al. 2010). This finding underscores the importance of nuanced education about the causes and risk factors of epilepsy. The finding that almost 68% of the participants believed that epilepsy is not contagious is encouraging, reflecting a degree of understanding about the nature of the disease. This aligns with the scientific consensus that epilepsy is not a contagious condition. This is an important finding, as it may directly reflect attitudes and practices that the society shows toward a patient experiencing an epileptic fit.

Despite the relatively high levels of awareness and understanding demonstrated by the participants, there were some major misconceptions identified. A noteworthy result was that about 26% of participants were of the view that ancestral sins may cause epilepsy, hence depicting a perceived spiritual basis of epilepsy among Ghanaians (Deegbe et al. 2019; Adjei et al. 2013). This perception, while suggesting a reduction in misconceptions (Baskind and Birbeck 2005), indicates that a belief in supernatural causes of epilepsy remains relatively high (Adjei et al. 2013). The perception of epilepsy as a hindrance to a happy life (88.7%), normal sexual life (51.1%), and education (84.8%) further reflects societal misconceptions and stigma surrounding the condition (Deegbe et al. 2019; Jacoby et al. 2005). This could potentially contribute to the reduced quality of life for individuals with epilepsy and emphasizes the need for interventions aimed at reducing stigma.

More than half (60.3%) of the participants believed that epilepsy is treatable, and 50.8% believed that individuals with epilepsy can be gainfully employed. While there is vast room for improvement, these findings indicate a certain level of understanding of the potential for individuals with epilepsy to lead normal, productive lives with appropriate treatment and support (Jacoby et al. 2005). Additionally, significant gender and age differences in knowledge about epilepsy exist. Similarly, students’ college affiliation also had a significant effect on their knowledge levels about epilepsy (Table 6). Students from health science‐related colleges like the College of Health and Allied Sciences, due to their foreknowledge of diseases such as epilepsy during lectures, tend to be more knowledgeable about the condition than their counterparts from non‐health science‐related colleges (Table 7). In summary, while there is a level of awareness and understanding about epilepsy among the participants, certain misconceptions still persist. There is a clear need for continued education and awareness campaigns to dispel these misconceptions and reduce the stigma associated with epilepsy.

The majority of the study participants had poor or inadequate knowledge about epilepsy, while only 7.1% had good or adequate knowledge (Figure 1). The remaining 27.1% demonstrated a moderate level of knowledge about the condition. Many factors could contribute to participants' knowledge levels about the condition. These include source of information, quality of teaching, socio‐economic status, parental involvement, personal motivation, and societal influence (Higgins and Simpson 2008; Sirin 2005; Jeynes 2007; Deci and Ryan 2000). If any of these factors were suboptimal in the population of participants, it might explain the higher percentage of participants with inadequate knowledge.

### 4.3 Attitudes Toward PLWE Among the Participants

The findings presented in Table 4 shed light on the attitudes of undergraduate students of UCC and traders at the Cape Coast's Abura Market in Ghana towards individuals with epilepsy. The participants' perception that people with epilepsy face discrimination in society (84.2%) highlights a pressing societal concern (Baker et al. 2000). This perception could be rooted in the numerous misconceptions and stigmas associated with epilepsy, which exists in societies worldwide, predominantly in developing countries (Fiest et al. 2014). On a more hopeful note, the majority of participants (77.3%) expressed their willingness to maintain friendships with individuals with epilepsy, suggesting a greater acceptance at a personal level. However, the results also reveal a dichotomy in attitudes toward romantic relationships and familial interactions with people living with epilepsy. Only 14.3% of participants were open to the idea of marrying someone with epilepsy. So, while a participant is ready to befriend a PLWE, sthe ame participant is unwilling to settle in marriage with the PLWE. Could it mean that such a friendship may not be genuinely constructed? The current finding is consistent with studies that point to stigma associated with marrying someone who has epilepsy (Jacoby 2002). Yet, more than two‐thirds of participants (67.1%) would let their children play with another child with epilepsy, suggesting a relative acceptance in non‐romantic, non‐familial relationships.

The participants' views on the educational placement of individuals with epilepsy were also quite revealing. About 43.2% opposed the idea of sending individuals with epilepsy to alternative/compensatory schools. This demonstrates a recognition of the potential of these individuals in mainstream education, aligning with the idea that epilepsy does not necessarily significantly limit cognitive or academic abilities (Fastenau et al. 2009). Meanwhile, 14.5% of the respondents were unwilling to share stationery with an individual living with epilepsy, which may reflect lingering misconceptions about the transmission of epilepsy.

**FIGURE 2:**
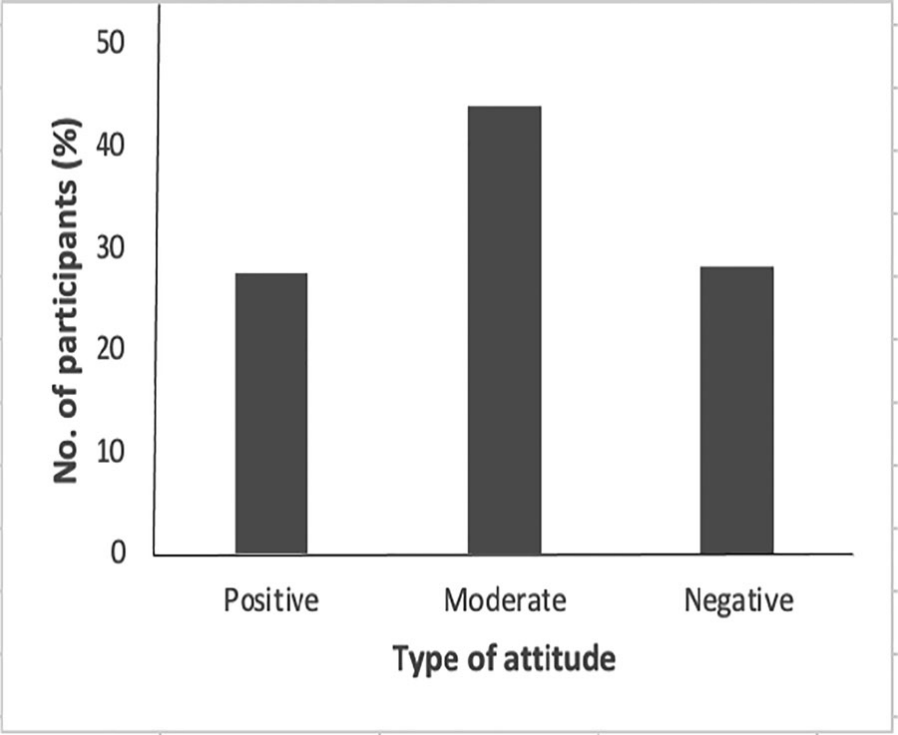
**Participants’ attitudes toward PLWE**. Almost the same number of participants indicated having negative and positive attitudes toward PLWE, that is, 28.4% and 27.6%, respectively. About half of the participants (44.0%) reported possessing a moderate attitude toward PLWE.

More than two‐thirds of the participants (68.8%) believed in the potential for success of individuals with epilepsy, and a large proportion (91.2%) expressed interest in learning more about the condition. This suggests a generally positive outlook and openness to further education or obtaining more credible knowledge about epilepsy (Bishop and Boag 2006). Also, significant differences were found in attitudes toward PLWE between students and traders as well as among students from the various colleges investigated (Tables 6 and 7), reflecting the potential of knowledge to influence attitudes. Overall, only 27.6% of the participants reported positive attitudes toward PLWE, suggesting that education and awareness‐raising efforts about epilepsy should be intensified to reshape societal attitudes (Figure 2). Meanwhile, it is refreshing that about half of the participants (44.0%) reported moderate attitudes toward PLWE; hence they are likely to change to more positive attitudes if the appropriate knowledge about the condition is obtained.

### 4.4 Practices of Students and Traders Toward PLWE

A high number of respondents (66.5%) reported they would put a spoon in the mouth of the patient, which is a widespread but incorrect practice in managing seizures (WHO 2019). This finding underscores the need for further education on proper first aid response to seizures, as this particular action can harm both the individual with epilepsy and the responder (Friedman 2011).

On a more positive note, the majority of participants indicated they would take appropriate actions during a seizure event: calling a doctor or nurse (84.8%), removing nearby objects (91.7%), and positioning the patient after the attack (90.0%). These are all considered beneficial practices (WHO 2019). This demonstrates that a large portion of students have acquired valuable knowledge on how to support individuals with epilepsy during a seizure event.

Nevertheless, some participants reported practices rooted in religious or cultural beliefs. More than half of the participants would start praying (51.3%); a smaller percentage will, however, apply anointing oil on the person (15.2%) or take the person to a religious place of worship, including a church, mosque, or shrine (19.4%). These findings align with prior research that has identified the influence of religious and cultural beliefs on attitudes and practices toward epilepsy (Elafros et al. 2013). Again, this reflects the religious nature of Ghanaians, particularly in identifying or managing complex, unexplained situations and events. Such practices may not be harmful per se, but they could potentially delay medical treatment or contribute to the perpetuation of stigmas surrounding epilepsy. Regarding practices towards someone experiencing an epileptic fit, gender (Pupillo et al. 2014) and college affiliation differences were obtained. The gender differences in practices toward PLWE are reaffirmed by the epilepsy knowledge differences found. The college affiliation may be reflective of acquired knowledge of appropriate practices toward a person having an epileptic attack.

**FIGURE 3:**
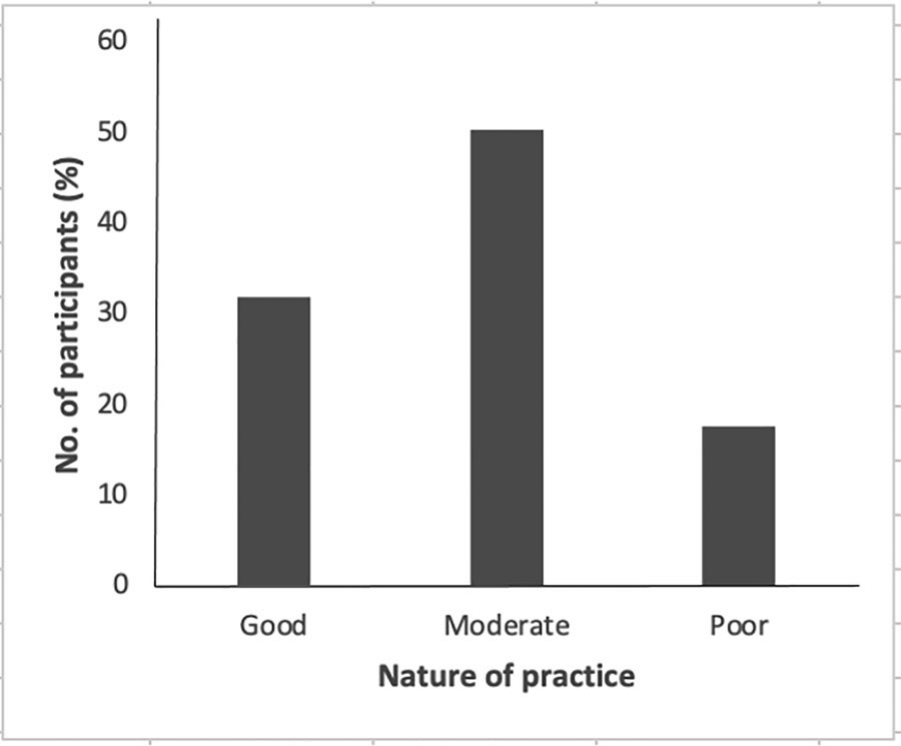
**Participants’ practices toward a person with an epileptic attack**. Half of the participants (50.4%) showed moderate/neutral practices toward a person experiencing an epileptic fit, while a lower number, that is, 94 (17.7%), revealed negative/poor practices. About a third of the participants, 170 (32.0%), indicated good/positive practices or first aid management for a person having an epileptic fit.

Just about a third of the participants (32.0%) were found to have good practices toward epilepsy, while 17.7% exhibited bad/poor practices (Figure 3). Quite encouragingly, the majority of them (50.4%) exhibited moderate practices toward the condition. These results suggest that a significant portion of the participants’ population have a relatively good understanding of how to manage and interact with individuals with epilepsy (Birbeck et al. 2007). However, the persistence of certain incorrect and potentially harmful practices highlights the importance of continued epilepsy education and awareness efforts to ensure appropriate responses during seizure events and to address any lingering misconceptions.

### 4.5 Determinants of Good Knowledge, Attitude, and Practices of the Participants Toward PLWE

Several factors that may influence students’ and traders’ knowledge, attitudes, and practices about epilepsy were also analyzed. The logistic regression model showed that, if unadjusted, gender, age, ethnicity, occupation, student level, college, prior knowledge of epilepsy, and positive/good attitudes and practices toward PLWE predicted a good or adequate level of knowledge about epilepsy (Table 8). However, when adjusted, only age, college, having ever seen an epileptic fit, and practices toward PLWE predicted adequate knowledge of epilepsy. Students’ college of affiliation significantly affected their knowledge base of epilepsy due to the contents of instructions or subject areas they are exposed to. Students from the College of Health and Allied Sciences demonstrated higher knowledge on epilepsy because they are studying health science‐related programs and most likely might have studied various diseases, including epilepsy. A similar reason could account for why students performed better on knowledge about epilepsy than the traders when considering the unadjusted odd ratios. The results showed that ethnic or cultural norms in the Ghanaian society might affect the level of knowledge people have about epilepsy.

Determinants of positive attitudes toward PLWE were subsequently investigated. Here, if unadjusted, demographic factors like occupation, level of students, and college, as well as ever seen or heard about epilepsy, adequate knowledge about epilepsy, and positive practices toward PLWE significantly predicted positive attitudes toward PLWE (Table 9). These findings could stem from the possession of more appropriate knowledge on epilepsy, as stated above regarding the determinants of good knowledge. Conversely, after adjusting the odds ratio, only ever hearing and seeing someone with an epileptic fit as well as possession of adequate knowledge of epilepsy significantly predicted good/positive attitudes about epilepsy. We also showed that with the unadjusted odds ratio, occupation, level of students, college, ever heard and seen someone with an epileptic fit, adequate knowledge about epilepsy, and positive attitude toward PLWE significantly predicted positive practices toward PLWE (Table 10). Just as we saw in the case of predictors of adequate knowledge after adjusting the odd ratios, for positive or good practices toward PLWE, we reported that having ever seen a person with an epileptic fit, a form of prior knowledge, is a significant determinant. All these predictors of adequate knowledge, positive attitude, and practices toward epilepsy have largely been confirmed by a number of previous studies (Fernandes et al. 2007; Pupillo et al. 2014). Based on the foregoing, prior knowledge or exposure to epilepsy, whether through personal experience or education, is a predominant predictor of appropriate attitudes and practices toward PLWE. Prior knowledge has been shown to significantly improve attitudes and practices toward epilepsy among children and respondents from other cultures, such as in Europe (Brabcová et al. 2017; McEwan et al. 2007). The current results underscore the potential value of educational interventions, focused on epilepsy, to foster more informed and positive KAP among people.

## 5 Limitations

The present study focused on persons who are not living with epilepsy. This was done to prevent any psychological trauma PLWE may experience while responding to epilepsy‐stigma‐associated questions. While this was appropriate, had we provided psychological support services for such participants (PLWE), we would have generated richer data in the study. This may be complemented by a qualitative approach, which may elicit important nuances underlying people's attitudes and practices toward PLWE. Similarly, the use of convenience sampling may affect the generalizability of our findings. The large presence of tertiary‐level students, aged 18–27 years, who participated in the study, could account for the wide range of confidence intervals for the upper and lower scores (Table 8), and this may affect the stability of this particular estimate.

## 6 Conclusion

The present study investigated the epilepsy‐related knowledge, attitudes, and practices (KAP) among university students and traders. Despite the majority of the respondents recognizing epilepsy as not a contagious disease or a result of ancestral sin, misconceptions also exist. Furthermore, the study demonstrated that participants' knowledge levels were insufficient, with less than a tenth having adequate knowledge about the condition. The attitudes and practices of the participants toward epilepsy were generally moderate, while only a third of them demonstrated positive attitudes and practices toward PLWE. After adjusting for odds ratio, we found that age, students’ college of affiliation, having ever seen an epileptic fit, and practice predicted adequate knowledge of epilepsy, while ever hearing and seeing someone with an epileptic fit as well as possession of adequate knowledge of epilepsy significantly predicted good/positive attitudes toward PLWE. To improve the KAP of participants toward PLWE and reduce societal misconception, discrimination, and stigmatization, the Ministry of Health and other relevant stakeholders should implement and intensify awareness campaigns and strengthen health promotion to educate the public about epilepsy, dispel misconceptions, and reduce the stigma associated with the condition. To do so, various communication outlets, including the mass media and community information centers, should be effectively used. Also, educational institutions should provide support systems for students with epilepsy, including access to medical services and academic accommodations, and promote an inclusive environment for all students.

## Author Contributions

N. A. O. O. contributed to the design of the study, data collection data, data analysis, and writing of the manuscript. A. M. U. contributed to data analysis and writing of the manuscript. F. T. D. contributed to the conceptualization and design of the study and the writing of the manuscript. K. A. contributed to the writing of the manuscript. D. L. E. led in the conceptualization and design of the study, contributed to data analysis and led in writing the manuscript. All authors approved the final version of the manuscript prior to submission.

## Funding

The authors have nothing to report.

## Conflicts of Interest

The authors declare no conflicts of interest.

## Data Availability

Data used in the study is available and can be provided upon formal request.
